# Social Media Use and Body Image Dissatisfaction Among University Students of Health Sciences in Saudi Arabia

**DOI:** 10.7759/cureus.74889

**Published:** 2024-12-01

**Authors:** Ihdaa J Abdulwahab, Jumana H Khouja, Noha A Alzahrani, Amina Bargawi

**Affiliations:** 1 Preventive Medicine Department, Saudi Board of Preventive Medicine, King Abdulaziz Medical City Jeddah, Jeddah, SAU; 2 Preventive Medicine, King Abdullah International Medical Research Center, Jeddah, SAU; 3 Preventive Medicine, King Saud Bin Abdulaziz University for Health Sciences, Jeddah, SAU

**Keywords:** body image dissatisfaction, gender, saudi arabia, social media use, university students

## Abstract

Background

Recently, social media has become an integral part of daily life, particularly among young adults. While these platforms offer numerous benefits, including communication, information sharing, and social connection, they have also been linked to a range of negative psychological consequences, including body image dissatisfaction. This study examined the prevalence of body dissatisfaction among health science students in Saudi Arabia, assessed the frequency and duration of social media use by gender, estimated the impact of social media use for >3 hours a day on body dissatisfaction, and identified the most commonly used social media platforms.

Methodology

Between December 2023 and June 2024, we conducted a cross-sectional study of students at King Saud Bin Abdulaziz University for Health Sciences, including all university colleges and branches in Jeddah, Riyadh, and Al-Ahsa. A survey was randomly sent to students’ email addresses by the research unit of each college. Chi-square and Fisher’s exact tests were used to test the association between two categorical variables.

Results

The 392 participants were between 18 and 25 years old; more than half (63%) were female and 95% were single. The majority (88%) were satisfied with their body image, whereas 11.2% were dissatisfied. Body dissatisfaction was significantly associated with gender (p = 0.038) and body mass index (p = 0.031). Approximately 87% reported spending ≤3 hours per day on social media, and 65% reported using social media six times or more. There were no associations between long duration and frequency of social media use and body dissatisfaction by gender (p > 0.05). The most prevalent social media platform was TikTok (63%), followed by Twitter (59%), and an equal percentage (53%) for WhatsApp, Instagram, and Snapchat.

Conclusions

This study indicates a low prevalence of body dissatisfaction among health science students. Increased duration and frequency of social media use were not significantly associated with body dissatisfaction. Focusing on body satisfaction among future physicians is crucial because it plays a significant role in shaping their patients’ perceptions of health and beauty. Future studies could expand on these findings by including larger and more diverse sample sizes. They could also explore the impact of different types of social media content on body dissatisfaction.

## Introduction

Social media (SM) is one of the most recent and popular forms of media worldwide. Over the past year, SM use has increased by more than 5% [1]. In January 2024, there were 5.04 billion SM users worldwide, representing 62.3% of the total population [1]. According to Global Digital Reports, the average time spent on SM daily is two hours and 23 minutes [1]. Specifically, Saudi Arabia has 35.1 million SM users who spend three hours per day on SM [2].

Several studies have suggested that increased SM use is related to increased body dissatisfaction (BD), depressive symptoms, social anxiety, and isolation, especially for those who follow celebrities, fitness inspiration content, and thin-ideal content [3-5].

BD is a widespread mental health issue that develops when an individual has continuous negative thoughts and feelings toward their body. Such individuals engage in behaviors such as frequent mirror checks, self-weighing, and avoiding public places. There are two aspects of body image disorder, namely, evaluative, which refers to the difference between the present and ideal body image, and emotional, which refers to an individual’s suffering due to this discrepancy [6]. It is increasingly being recognized as a serious risk factor for mental health problems. This leads to low self-esteem, social isolation, obsession with looks, depression, and harmful weight gain control behaviors, such as avoidance of physical activity, addiction to exercise, substance use, and cosmetic procedures, which are some of the health consequences of BD [5,7,8].

A 15-year longitudinal study from mid-adolescence to adulthood demonstrated increases in BD within the 15 years, which was attributed to weight gain; additionally, nearly 95% of individuals from childhood through adulthood reported relatively constant BD [9]. BD is prevalent among both genders, particularly women, ranging from 24% to 46% among girls and 12% to 26% among boys [10-12]. In the United Kingdom, SM images caused one in five individuals to feel concerned about their body image [13].

Regarding the prevalence of BD in Saudi Arabia, one study found the prevalence of BD among university students to be 33.5% among females and 21.40% among males [14]. A similar study among Saudi women from 12 fitness centers in Riyadh reported that 87% were dissatisfied with their body shapes. This included 68% of average-weight women, with approximately 40% of women having an incorrect perception of their physical form [15]. By contrast, in a cross-sectional study of undergraduate students, only 28.9% of students revealed a high degree of dissatisfaction, whereas 71.1% had a low level of dissatisfaction [16]. Several studies in SA have found that SM use for three to four hours, short duration of sports club membership, high socioeconomic status, high body mass index, and being married are associated with higher levels of BD [14-16].

Saudi Arabia’s use of SM has grown dramatically and become an integral part of the lifestyle. Physicians can also act as role models for patients by influencing their perceptions and decisions. For example, patients with a healthy appearance should be convinced to avoid cosmetic surgery on the face or body. However, to our knowledge, no studies have been conducted in Saudi Arabia to examine the prevalence of BD among future physicians. The objectives of this study were to examine the prevalence of BD among health science students, assess the frequency and duration of SM use by gender, estimate the impact of SM use for >3 hours a day on BD, and identify the most common SM platforms.

## Materials and methods

Study design and setting 

In this cross-sectional study, data were collected between December 2023 and June 2024 from King Saud Bin Abdulaziz University for Health Sciences (KSAU-HS), including all university colleges and branches in Jeddah, Riyadh, and Al-Ahsa.

Ethical considerations

Ethical approval was obtained from the institutional review board of the King Abdullah International Medical Research Center (KAIMRC) (approval number 2919/23). Consent was obtained and mentioned in the introduction section of the questionnaire. During the study, confidentiality and privacy were maintained.

Participants

The participants were selected based on the following criteria: students at KSAU-HS, including all university colleges (the college of medicine, dentistry, pharmacy, science and health professions, applied medical sciences, nursing, and public health and health informatics) from all branches of the university in the Kingdom (Jeddah, Riyadh, and Al-Ahsa). The participants’ ages ranged from 18 to 25 years. Only iPhone users were selected because they could obtain the average time spent on SM from the iPhone screen time settings. Participants were excluded if they did not complete the questionnaire; were non-iPhone mobile users, such as Android users; and were medical residents or doctors.

Sample size and sampling technique

The sample size was calculated using the Raosoft sample size calculator (Raosoft Inc., n.d.). Population size was based on a population census conducted by the Saudi General Authority for Statistics in 2023. The accepted margin of error was 5%, confidence interval was 95%, and power was 80%. The minimum sample size required was 385 participants.

This study utilized a stratified random sampling method in which 500 participants were divided into the following three strata: 200 from Riyadh, 200 from Jeddah, and 100 from Al-Ahsa. For each stratum, we used a simple random sampling method by randomly sending emails to college students.

Data collection methods and measurements

An electronic questionnaire was created using Google Forms (Google LLC, California, USA) and distributed via student university emails. A total of 437 responses were recorded during the survey period. Of these, 45 respondents were Android users, residents, or did not complete the questionnaire. As a result, 392 responses, obtained from students using iPhones, were analyzed.

Demographic Information

The sociodemographic questions in the questionnaire included information regarding the students’ age, gender, marital status, weight, height, and the name of the university branch and college.

Body Mass Index

The weight and height of the participants were recorded to calculate the body mass index (BMI). The result of the BMI calculation using the formula (weight (kg)/height^2^ (m)) multiplied by 10,000 was then split into six different categories: (1) <18.5 kg/m^2^ (underweight), (2) 18.5-24.9 kg/m^2^ (normal weight), (3) 25-29.9 kg/m^2^ (overweight), (4) 30-34.9 kg/m^2^ (obesity class 1), (5) 35-39.9 kg/m^2^ (obesity class 2), and (6) 40 kg/m^2^ (obesity class 3).

The Body Image Satisfaction Scale

A valid and reliable scale adapted from Tajuddin et al. [17] was used to measure satisfaction with body image. The scale was self-reported on a five-point Likert scale (strongly agree, agree, neutral, disagree, and strongly disagree). It consisted of 29 statements, with 12 positive and 17 negative statements. These statements were distributed across the following three dimensions: cognitive, emotional, and behavioral. A score of 86 or below indicated BD, whereas a score of 87 or above indicated body satisfaction.

Duration and Frequency of Social Media Use

To explain how the time spent on SM was calculated, an image was captured from an iPhone screen time setting. This image was attached to an online survey. Students were asked to record the amount of screen time they spent on SM each week on their mobile devices. After dividing the average weekly recording time by 7, we determined the average daily recording time. As far as SM frequency was concerned, it was categorized as daily, with the following three options: two to three times, four to five times, or over six times. Students chose from a variety of popular SM platforms, including WhatsApp, Twitter, Telegram, Instagram, TikTok, and Snapchat.

Data analysis

Data entry and analysis were performed using the SPSS statistical software package version 21.0 (IBM Corp., Armonk, NY, USA). Descriptive statistics for categorical variables were determined and presented as frequencies and percentages. Inferential statistics used the chi-square and Fisher’s exact tests to test the association and/or the difference between two categorical variables. The significance level was set at p-values <0.05.

## Results

Between December 2023 and June 2024, 392 students who met the inclusion criteria responded to the questionnaire. Approximately 171 (44%) of the university students were from Jeddah, 162 (41%) were from Riyadh, and 59 (15%) were from Al-Ahsa. We received responses from all health science colleges, including the colleges of medicine, dentistry, pharmacy, science and health professions, applied medical sciences, nursing, and public health and health informatics. All students were undergraduates or interns aged between 18 and 25 years. Of all participants, 247 (63%) were female, and 372 (95%) were single. Students were mostly satisfied with their body image (348, 88%), whereas 44 (11.2%) were dissatisfied with their body image. In terms of BMI, 54 (14%) were underweight, 206 (53%) were normal, 82 (21%) were overweight, 31 (8%) were in obesity class 1, 13 (3%) were in obesity class 2, and 6 (1%) were in obesity class 3. Table 1 summarizes the students’ general sociodemographic characteristics in relation to body image satisfaction.

**Table 1:** Sociodemographic characteristics of participants. *: Significant at p-values less than 0.05; **: row percent; ^: Fisher’s exact test.

Variables		Total	Body dissatisfaction scale				X^2^	P-value
		(N = 392)	Dissatisfied		Satisfied			
			44 (11.2%)		348 (88.8%)			
			N	%**	N	%**		
Age group	18–21	260 (66%)	28	11%	232	89%	0.161	0.689
	22–25	132 (34%)	16	12%	116	88%		
Gender	Female	247 (63%)	34	14%	213	86%	4.326	0.038*
	Male	145 (37%)	10	7%	135	93%		
Marital status	Single	372 (95%)	43	12%	329	88%		0.713^^^
	Married	20 (5%)	1	5%	19	95%		
University branch	Jeddah	171 (44%)	15	9%	156	91%	1.846	0.397
	Riyadh	162 (41%)	21	13%	141	87%		
	Al-Ahsa	59 (15%)	8	14%	51	86%		
College name	Medicine, dentistry, pharmacy	140 (35%)	15	11%	125	89%	5.864	0.118
	Science and health professions	117 (30%)	12	10%	150	90%		
	Applied medical sciences	81 (21%)	6	7%	75	93%		
	Nursing, public health, and health informatics	54 (14%)	11	20%	43	80%		
Body mass index	Underweight	54 (14%)	5	9%	49	91%	4.63	0.031*
	Normal	206 (53%)	19	9%	187	91%		
	Overweight	82 (21%)	10	12%	72	88%		
	Obesity class 1	31 (8%)	6	19%	25	81%		
	Obesity class 2	13 (3%)	2	15%	11	85%		
	Obesity class 3	6 (1%)	2	33%	4	67%		

BD was significantly associated with gender (p = 0.038) and BMI (p = 0.031); however, other sociodemographic factors, such as age, marital status, university branch, and college name, were not statistically significant (p > 0.05).

Table 2 shows that most students (340, 87%) spent less than or equal to three hours using SM daily. Half of all students (255, 65%) used SM platforms at least six times a day. However, the duration or frequency of SM engagement was not significantly associated with BD (p > 0.05).

**Table 2:** Duration and frequency of using social media (SM) platforms. *: Row percent.

Variables		Total	Body dissatisfaction scale				X^2^	P-value
		(N = 392)	Dissatisfied		Satisfied			
			44 (11.2%)		348 (88.8%)			
			N	%*	N	%*		
Number of hours spent on SM daily	≤3 hours	340 (87%)	38	11%	302	89%	0.015	0.904
	4–6 hours	48 (12%)	6	12%	42	88%		
	7–9 hours	4 (1%)	0	0%	4	100%		
Number of times SM is used daily	3 times or less	61 (16%)	7	11%	54	88%	0.002	0.899
	4–5 times	76 (19%)	8	10%	68	89%		
	6 times or more	255 (65%)	29	11%	226	89%		

**Figure 1:**
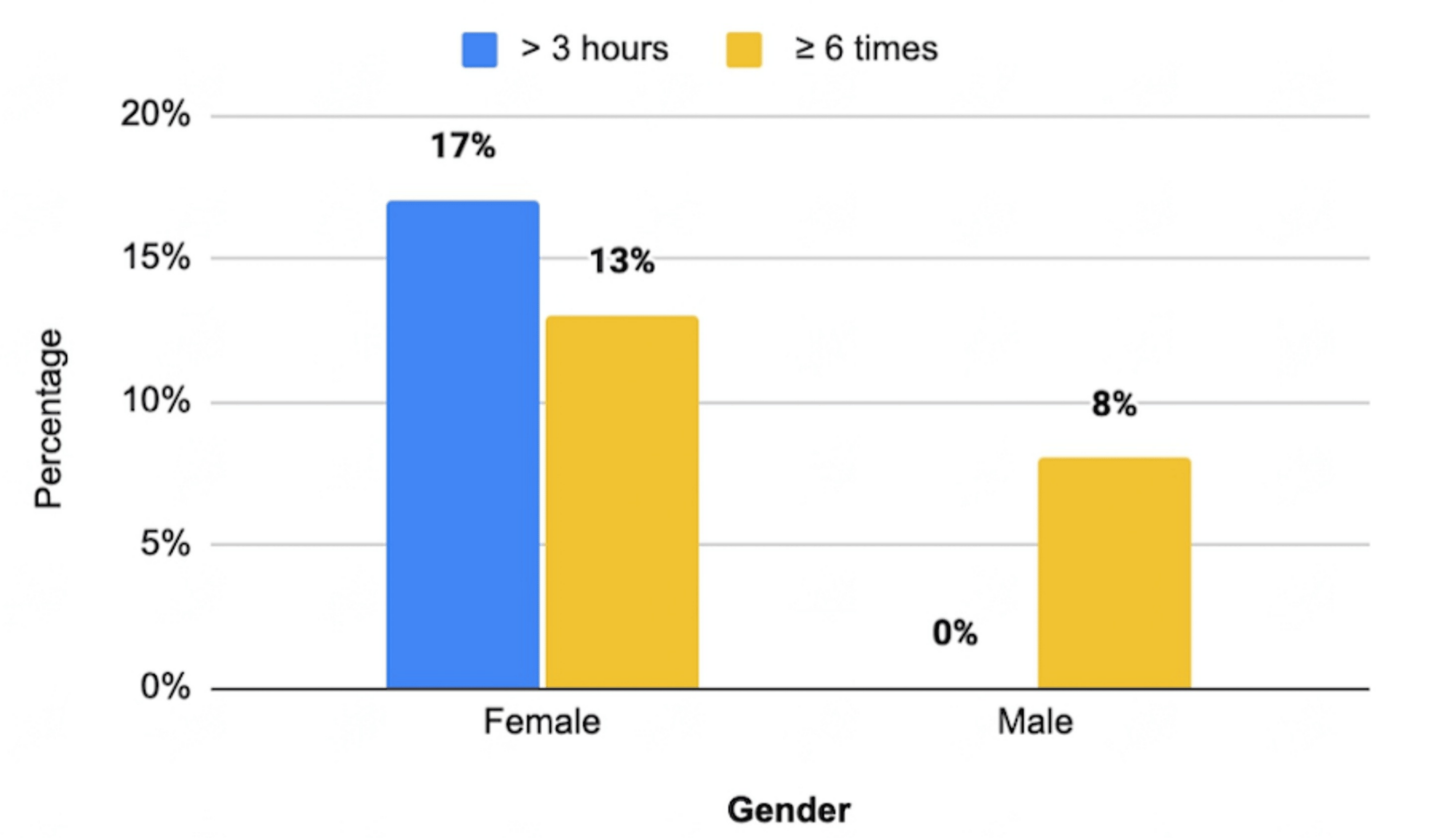
Duration and frequency of daily social media use among males and females with body dissatisfaction. P > 0.05.

Figure 1 compares the use of SM for >3 hours daily and the frequency of engaging in SM among males and females with body image dissatisfaction. Females with BD who used SM for over three hours accounted for 6 (17%), whereas males accounted for 0%. Females (13.5%) used SM platforms six times or more, whereas males (8%) used SM platforms less. The higher proportion of females in our sample may explain this finding. There were no associations between SM duration, frequency, and BD between genders (p > 0.05).

**Figure 2:**
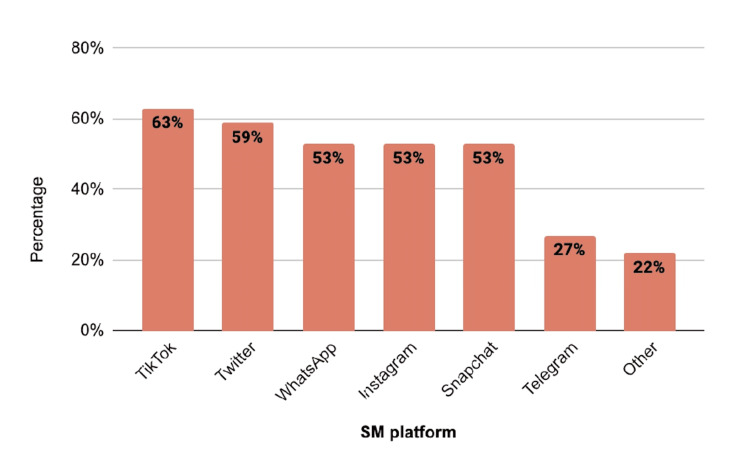
Most commonly used social media (SM) platforms. Twitter and body dissatisfaction (P = 0.021).

Figure 2 shows that the most common SM platform prevalent among the students was TikTok at 248 (63%), followed by Twitter at 231 (59%), and an equal percentage of WhatsApp, Instagram, and Snapchat at 208 (53%). The least prevalent was the use of Telegram at 107 (27%).

## Discussion

This study assessed the prevalence of BD and its association with SM use among university health science students in Saudi Arabia. The findings provided insights into the prevalence of BD, its association with the duration and frequency of SM use, and common SM platform use among students.

As reported in our study, the prevalence of BD was 14% among females and 7% among males. In a review conducted by Martini et al. [18], BD prevalence was between 18% and 56.6% in both genders. In another review conducted by Fiske et al. [19] in the United States, the prevalence of BD varied widely between 11% and 72% among women and from 8% to 61% among men. The prevalence observed in the present study was at the lower end of this range. This could be attributed to health science students having a positive perception of their body image. In addition, they may be more aware of it than others are. Through education, children can recognize normal physiological and anatomical body structures. Consequently, they are more likely to make healthy lifestyle choices that contribute to their overall physical and mental well-being.

BD was more common among females than males. This is consistent with previous studies conducted among university students in Saudi Arabia, which found a higher prevalence of BD among females than males [14,16]. This could be attributed to the gender stereotypes created by family, media, peers, and Western cultures. Men are portrayed as strong and muscular, whereas women are portrayed as thin and attractive [7]. Recently, it was found that the incidence of BD increases with age among both women and men [20]. Hence, in both traditional and Western cultures, young men tend to favor masculinity and an ideal manly shape [21]. Across studies, we observed a wide range of BD prevalence. This could be due to the use of different methods for assessing BD, such as scores and questionnaires [18]. This study used a newly developed scale to assess dissatisfaction.

This study found that an increased BMI was significantly associated with BD (p = 0.031), indicating that obese students were generally dissatisfied with their bodies. Our findings, in accordance with those of a previous review, indicated that BMI is a strong indicator of BD [7]. Owing to the growing prevalence of obesity in Saudi Arabia, this factor may be a significant predictor of dissatisfaction among younger generations.

Additionally, we found that BD was not related to the long duration or frequency of SM use. More than half the students reported using SM at least six times per day. However, no significant relationship was identified between the frequency of SM use and BD (P ≥ 0.05). Conversely, several studies have demonstrated that individuals with BD have a higher level of engagement and frequency of SM use than those who are satisfied [3,22]. Tadena et al. [22] examined the influence of SM affinity on BD. They found that individuals with SM affinity are 16 times more likely to develop BD. Similarly, a mixed systematic review showed that significant levels of SM involvement or exposure to image-related information were related to higher levels of BD, dieting, food restriction or binging, and healthier food choices. Consequently, viewing ideal pictures of celebrities, friends, fashion and fitness, food, talking about body fat, and seeking reassurance may have increased these risks [23]. Additionally, spending more time on SM increases the risk of BD [16,24,25]. Despite this, our findings did not indicate a significant relationship between SM use for >3 hours and BD (p > 0.05). This could be attributed to the fact that the majority of our sample spent less than or equal to three hours per day on SM. It is also possible that health science students have adequate awareness of their body image and use SM in a controlled and rational manner. Moreover, long study hours and busy schedules prevent them from spending extended periods on social media. This discrepancy may also be due to the lack of standard measurement methods for SM, as other studies have relied on questionnaires including SM scores, affinity scores, engagement, and addiction scales, all of which are subjective. In this study, objective measures based on mobile records were used to reflect the real time spent on SM.

In terms of SM use and BD between genders, neither frequency nor duration were significantly associated (p > 0.05). This finding is contrary to a systematic review of females who used SM for more than two hours daily, which found that they were more likely to report BD (OR = 2.02; 95% CI = 1.30-3.16). Moreover, they tended to see themselves as overweight (RRR = 2.20; 95% CI = 1.34−3.60) when compared with those who used SM infrequently or never [24].

Our study revealed that TikTok was the most common SM platform used by students. This corresponds to global usage because TikTok was the first platform to record the largest amount of time spent on SM [1]. In our study, TikTok was followed by Twitter, WhatsApp, Instagram, and Snapchat, each with equal percentages. Telegram use was the least prevalent. Despite the possibility that the participants could use more than one SM platform, the percentage of BD was approximately the same across all SM platforms. However, Twitter was the only significant SM platform associated with BD (p = 0.021). Visual platforms (e.g., Instagram, Snapchat, and TikTok) are more destructive to body image, which can result in BD, than textual platforms (e.g., Facebook) [4].

Furthermore, idealized self-presentation is a significant factor because adolescents are highly dependent on peer feedback; for example, the number of “likes” and comments on photographs has a substantial impact on their mental health, including their body image [26]. In addition, SM’s 24/7 access to one’s and other people’s photographs, as well as the reactions to those images, create an ever-present appearance culture [26].

Limitations

This study had a few limitations. First, the number of hours spent on SM may not be a true reflection of reality because students usually use multiple devices to study, such as iPads or laptop computers. Second, to investigate the impact of different types of SM content on BD, we did not ask about the content viewed on SM platforms. Third, significant influencing factors need to be assessed, including parents’ education level, income level, and mental health issues, including anxiety and depression, eating disorders, as well as lifestyle factors, such as physical activity and dietary habits.

Future studies could expand on this study’s findings by including a larger and more diverse sample size from various universities and different age groups, such as adolescents. This would enhance the generalizability of the results. Future studies should also include non-iPhone users. A longitudinal case-control study would provide a better understanding of the risk factors and temporal associations between SM use and BD. Additionally, qualitative assessments, such as in-depth interviews or focus groups, could provide deeper insights into the underlying reasons behind BD and the specific role SM plays in shaping these perceptions. Researchers could also explore the impact of different types of SM content, such as fitness influencers or beauty standards, on body image. This would enable them to understand the relationship between SM use and BD better, collaborate with policymakers and educators, integrate media literacy education into school curricula to teach critical thinking skills, help students identify unrealistic beauty standards, promote body positivity and self-acceptance in classrooms and school environments, and Offer evidence-based interventions, such as cognitive-behavioral therapy and mindfulness-based interventions, to address body image concerns.

## Conclusions

This study indicates a low prevalence of BD among health science students. The increased duration and frequency of SM use were not significantly associated with BD. Female students were more likely to experience BD than male students. In addition, BMI was one of the most influential factors associated with BD. Body image awareness is crucial in the medical field because healthcare professionals play a significant role in shaping patients’ perceptions of health and beauty. This awareness enables them to provide better psychological support and guidance to patients struggling with body image issues, ultimately leading to improved overall health outcomes.
